# Upcycling distillery waste: N, P, Fe Co-doped porous biochar from excess sludge of sauce-flavor liquor for high-performance supercapacitors

**DOI:** 10.1039/d5ra02656c

**Published:** 2025-06-11

**Authors:** Suxing Luo, Jiang Li, Meizhi Yang, Ping Zeng, Yuanhui Wu

**Affiliations:** a School of Chemistry and Chemical Engineering, Zunyi Normal University Zunyi China suxingluo@126.com yhwull@126.com; b Special Key Laboratory of Electrochemistry for Materials of Guizhou Province Zunyi China; c College of Chemistry and Chemical Engineering, Shanxi Datong University Datong 037009 China; d Department of Research and Development, Guizhou Open University Guiyang China

## Abstract

Enhancing supercapacitor performance by incorporating heteroatoms into biochar represents a fascinating strategy. Our study presented an uncomplicated process for creating porous biochar simultaneously doped with nitrogen (N), phosphorus (P), and iron (Fe). The source material was excess sludge from sauce-flavor liquor production, which conveniently provided high initial N and Fe content for the resulting biochar. The phosphorus originated from phytic acid, while gentle activation was achieved using potassium carbonate (K_2_CO_3_). We found that adjusting the K_2_CO_3_ proportion and activation heating allowed precise control over the final material's characteristics, including its pore network, surface chemistry, plus the ultimate N, P, and Fe levels. Through electrochemical assessment, the N, P, Fe/BC-700-2 sample demonstrated superior performance, exhibiting a specific capacitance up to 141.7 F g^−1^ when tested at a 1 A g^−1^ current density in 6 M KOH electrolyte. This efficacy stemmed from several contributing elements: a large specific surface area (BET, 824.5 m^2^ g^−1^), abundant structural imperfections coupled with extensive mesoporosity, and advantageous synergistic effects arising from N, P, Fe co-incorporation. This investigation demonstrates that transforming sauce-flavor liquor excess sludge (SFLS) into heteroatom-enriched biochar not only offers significant promise for developing advanced supercapacitor electrode materials but also provides a sustainable pathway for valorizing problematic industrial waste.

## Abstract 2

Enhancing supercapacitor performance by incorporating heteroatoms into biochar represents a fascinating strategy.

## Graphical abstract

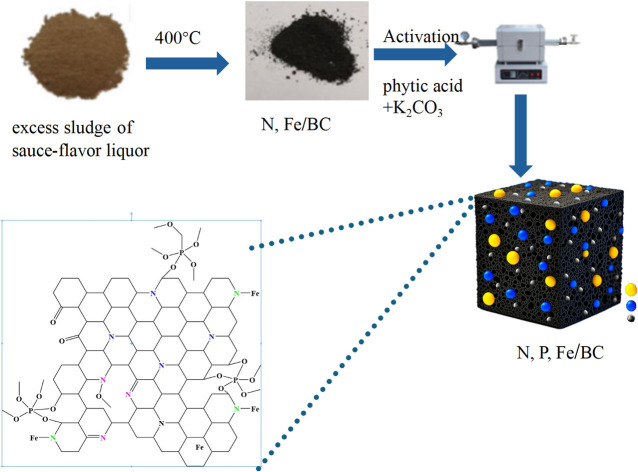

## 1 Introduction

The burgeoning global energy demand and the environmental impact of fossil fuels necessitate a paradigm shift towards sustainable energy storage solutions.^1,2^ Electric double-layer capacitors (EDLCs), or supercapacitors, are at the forefront of this transition, prized for their rapid charge–discharge capabilities, exceptional cycle stability, and high-power density.^3^ However, their broader market penetration is currently constrained by substantial manufacturing costs and relatively modest energy densities.^4^ Consequently, the design and synthesis of cost-effective electrode materials with superior electrochemical performance remain a critical research imperative.^5^

Biochar, derived from pyrolyzed biomass, has emerged as a promising candidate for supercapacitor electrodes due to its inherent porous architecture, large potential surface area, and low production cost.^6,7^ Yet, pristine biochar often exhibits limited electrochemical activity.^8,9^ It is well-established that heteroatom doping-incorporating elements like nitrogen (N), sulfur (S), phosphorus (P), or oxygen (O) can significantly enhance energy storage performance by introducing pseudocapacitance and improving surface wettability and electronic conductivity.^4,10^ While various strategies exist for heteroatom incorporation, utilizing biomass precursors intrinsically rich in desired elements offers a more streamlined, economically viable, and environmentally benign pathway.^11,12^ While biomass-derived carbons, such as the N, P, Fe co-doped biochar investigated herein, offer sustainable routes to supercapacitor electrodes, significant research efforts also focus on inorganic materials including transition metal oxides,^13,14^ sulfides,^15,16^ and their composites with conductive scaffolds like graphene^17,18^ to achieve high electrochemical performance.

Guizhou province, a major hub for Chinese liquor production (projected 600 000 m^3^ by 2025), generates substantial volumes of sludge-approximately 1–2% of the 60 m^3^ water consumed per m^3^ of liquor.^19^ This “sauce-flavor liquor sludge” (SFLS) presents not only a pressing environmental disposal challenge^20^ but also an overlooked opportunity for valorization. Improper disposal can contribute to soil and water contamination due to its high organic load and potentially leachable component. Distinct from municipal sludge, SFLS is characterized by consistently high organic matter, elevated nitrogen content, and notably, significant iron (Fe) concentrations stemming from the Fe(OH)_3_ used as a flocculant in its processing, all while maintaining low levels of heavy metals. These inherent compositional attributes render SFLS an exceptionally promising, yet largely untapped, precursor for *in situ* N and Fe co-doped biochar.^21,22^

**Scheme 1:**
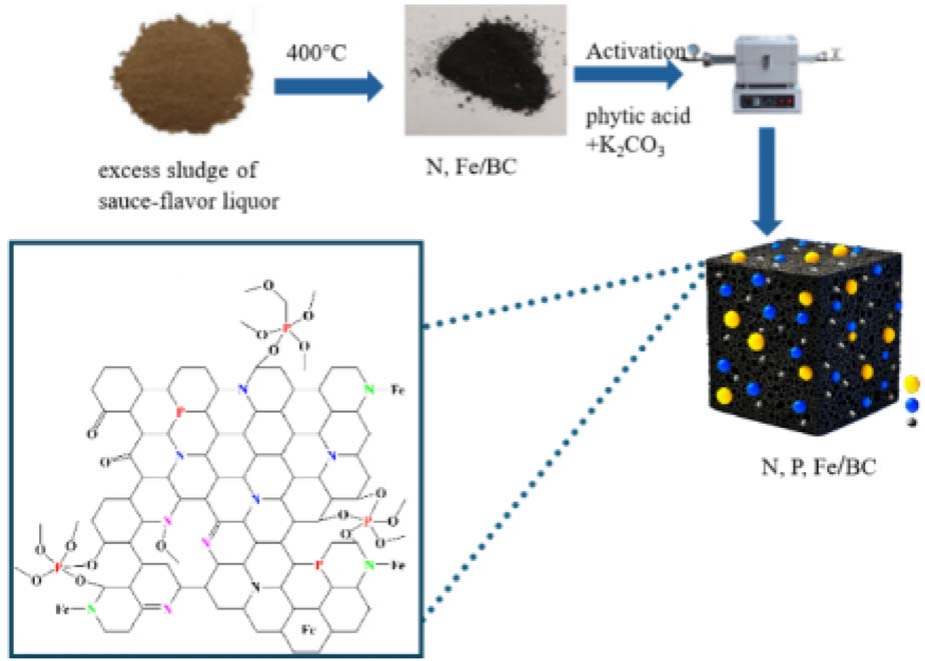
Schematic illustration of the preparation of porous biochar co-doped with N, P, and Fe, derived from excess sludge of sauce-flavor liquor.

While various biomass and waste streams have been investigated for biochar production, and sludge-derived biochars are gaining traction, a critical research lacuna exists. To address this gap, the present study pioneers the synthesis of novel N, P, Fe tri-doped porous biochar materials derived from SFLS (Scheme 1), offering a sustainable and cost-effective alternative to conventional carbon precursors often reliant on virgin or fossil-fuel-based resources. Most studies focus on agricultural wastes or generic sludges, often overlooking the unique elemental fingerprint that industrial byproducts like SFLS can offer for tailored heteroatom doping.^5,12^

Our strategy ingeniously integrates the self-doping potential of SFLS (N and Fe) with an exogenous phosphorus source, phytic acid (Scheme 1). Critically, phytic acid serves a dual role: not only as a P-dopant but also as an *in situ* activating/templating agent during high-temperature pyrolysis, promoting hierarchical pore development and defect creation. This is further complemented by K_2_CO_3_ activation, a milder and eco-friendlier chemical agent,^4^ to meticulously tailor the porous architecture. We systematically investigate the influence of K_2_CO_3_ dosage and activation temperature on the material's physicochemical properties and resulting electrochemical performance. This work, therefore, presents a novel and sustainable ‘waste-to-wealth’ strategy, transforming a problematic industrial byproduct into a high-value electrode material, contributing to both greener energy storage solutions and circular economy principles.^5,23^

## 2 Materials and methods

### 2.1 Materials and reagents

The excess sludge from sauce-flavor liquor was obtained from Guizhou Zhenjiu brewing Co., Ltd, located in China. The excess sludge underwent drying overnight at 105 °C. Subsequently, the dried sludge was mechanically crushed and then passed through a 100-mesh sieve with an aperture size of 0.15 mm. The organic matter content of the sample is 8.3% and the nitrogen content is 4.5%, which is much higher than the content in excess sludge from urban sewage treatment plants (organic matter 2.6–7.0%, nitrogen 0.5–4.0%). Phytic acid (50% solution), hydrochloric acid (HCl), and potassium carbonate (K_2_CO_3_) were obtained from Aladdin Industrial Co. (Shanghai, China). All chemicals were used without any additional processing.

### 2.2 Fabrication of N, P, Fe co-doped porous biochar

Excess sludge was first pyrolyzed at 400 °C for 2 h in an argon atmosphere, heated at a rate of 5 °C min^−1^, which yielded sludge biochar. Following this, 3 g of the produced biochar was combined with three milliliters of a 50 wt% phytic acid solution in twenty milliliters of ultrapure water; subsequently, varying quantities of K_2_CO_3_ (0, 3, 6, and 9 g) were introduced to examine the influence of activator loading on the material's surface area and pore characteristics. Afterward, the desiccated mixture underwent activation through heating to 600 °C and maintaining that temperature for 4 h within an argon atmosphere. To assess the influence of activation temperature, mixtures containing a specific K_2_CO_3_ quantity were subjected to pyrolysis. Separate treatments were conducted at 600, 700, 800, and 900 °C, each lasting 4 h within an argon environment. Afterwards, the resulting solid material was purified; this involved washing with 1 mol L^−1^ HCl followed by multiple rinses using pure water until the pH became neutral. The finalized samples received the designation N, P, Fe/BC-*T-n*. Here, ‘*T*’ signifies the particular activation temperature employed (600, 700, 800, or 900 °C), and ‘*n*’ denotes the simplified mass ratio of K_2_CO_3_ to biochar (represented as 0, 1, 2, or 3).

### 2.3 Characterization

To characterize the biochars, we examined their morphology and elemental distribution using scanning electron microscopy (SEM, TESCAN MIRA LMS). The chemical nature of the material's surface was explored *via* X-ray photoelectron spectroscopy (XPS, Thermo Scientific K-Alpha). Nitrogen (N_2_) adsorption–desorption isotherms, acquired using a Quantachrome EVO surface area and pore size analyzer, provided insights into specific surface area and porosity. Moreover, Raman spectroscopy, performed using an XDR Raman microscope from Thermo Fisher Scientific, was used to examine the structural attributes of the biochar samples. These spectra were obtained utilizing 455 nm laser excitation across the 800 to 2500 cm^−1^ spectral range.

### 2.4 Electrochemical measurements

To construct the working electrodes needed for electrochemical analysis, we first prepared a slurry. This involved combining the synthesized N, P, Fe co-doped biochar material (the active component) with acetylene black (for conductivity) and polyvinylidene difluoride (PVDF, as a binder) using an 8 : 1 : 1 mass ratio. *N*-Methyl pyrrolidinone (NMP) served as the solvent to facilitate mixing. This homogenous slurry was subsequently coated evenly onto current collectors. After coating, the electrodes underwent a drying process at 80 °C lasting 24 hours. Finally, each prepared electrode received compression treatment at 2 MPa pressure, and its mass was accurately determined for subsequent use in calculations. The performance attributes of the N, P, Fe multi-doped biochar electrodes were explored utilizing a 6 M KOH solution as the electrolyte. All tests utilized a two-electrode cell arrangement connected to an electrochemical workstation (CHI760D, Shanghai Chenhua). For this setup, one prepared electrode on its current collector acted as the working electrode, a platinum mesh as the counter electrode. Cyclic voltammetry measurements were taken between 0 and 1 V, with sweep rates ranging from 5 mV s^−1^ to 50 mV s^−1^. For the assessment of charge storage and release characteristics, galvanostatic cycling tests were conducted, employing current loadings ranging from 1 to 10 A g^−1^. Furthermore, electrochemical impedance measurements were carried out within a frequency span of 0.01 Hz up to 100 kHz, utilizing a minor voltage oscillation of 5 mV. Ultimately, the calculation of specific capacitance relied on the discharge portion of the galvanostatic curves, following the ensuing equation:

1 *C*_s_ = *I*Δ*t*/*m*Δ*V*

where *I* (A) represents the current applied during the discharge phase; Δ*t* (s) the total time duration for discharging; *m* (g) refers to the combined mass of the active material present on both electrodes; and Δ*V* (V) indicates the potential range (voltage window) across which the discharge occurs.

## 3 Results and discussion

### 3.1 Characterization of the materials

#### 3.1.1 The effect of K_2_CO_3_/BC mass ratio

**Fig. 1:**
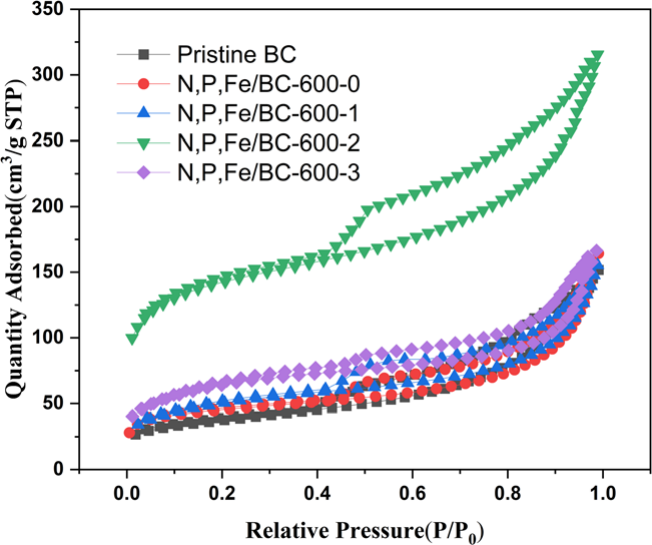
N_2_ adsorption–desorption isothermal curves of pristine BC and N, P, Fe/BC-600-*n* (*n* = 0, 1, 2, 3) at 77 K.

A biochar's specific surface area and its pore dimensions critically influence its capacity for energy storage.^24^ The mechanism underlying ion adsorption and subsequent release,^25–27^ specifically happening at the electrode–electrolyte junction, is critically influenced by these particular structural characteristics.^28,29^ Analyses of nitrogen adsorption and desorption took place at 600 °C on the synthesized pristine BC and N, P, Fe/BC samples, varying the K_2_CO_3_/BC mass ratio, to gain deeper insights into their specific surface area and pore characteristics. The N, P, Fe/BC adsorption–desorption isotherms displayed type-IV behavior according to the IUPAC classification (shown in Fig. 1) and the BET results, suggesting that mesopores were the predominant pore structures.^30^ When the relative pressure reached 1.0, a noticeable surge in nitrogen adsorption occurred, suggesting the existence of macropores. According to the previous reports, mesopores are vital for boosting ion movement by shortening travel distances and lowering electrolyte resistance. Additionally, larger macropores play a part by bolstering the electrical double-layer capacitance.^31^ Meanwhile, Hysteresis loops of the H3 type, detected within the relative pressure range of 0.4 to 1.0, suggest the presence of slit-like pores or extensive sheet-like arrangements with wide openings.^32^ According to Table 1, the BET specific surface areas and pore volumes of all samples showed an increase in BET specific surface area from 40.1 m^2^ g^−1^ to 133.0 m^2^ g^−1^ at 600 °C due to the addition of the P source. This implied that the inclusion of phytic acid not only facilitated P doping onto the biochar surface but also functioned as an activating agent, leading to a more developed pore structure within the biochar.^33,34^ H^+^ dissociated from phytic acid mostly attacked the C–O bond in biomass, accelerating condensation of alcohol or ether moieties, resulting in the formation of porous structure. Moreover, phytic acid could protected the carbon skeleton from pore collapse.^35^ As the K_2_CO_3_/BC mass ratio was elevated from 0 to 3, the BET specific surface areas of the resulting materials initially expanded before contracting, peaking when the mass ratio reached 2. The highest specific surface area (467.3 m^2^ g^−1^) and total pore volume were displayed by N, P, Fe/BC-600-2, implying that a K_2_CO_3_/BC mass ratio of 2 best facilitated the creation of a more porous structure in the biochar. While the K_2_CO_3_/BC mass ratio increased to 3, the surface area was decreasing to 229 m^2^ g^−1^, probably due to collapsing of the structure to increase the pore size with the aid of an alkali effect.^36^

**Table 1:** BET data of pristine BC, N, P, Fe/BC-600-*n* (*n* = 0–3)

Sample	*S*_BET_ (m^2^ g^−1^)	Total pore volume (cm^3^)	Average pore size (nm)
Pristine BC-600	50.1	0.1133	8.528
N, P, Fe/BC-600-0	133.0	0.2353	7.06
N, P, Fe/BC-600-1	179.58	0.2409	5.36
N, P, Fe/BC-600-2	467.3	0.4109	3.51
N, P, Fe/BC-600-3	229.0	2.58	4.54

**Fig. 2:**
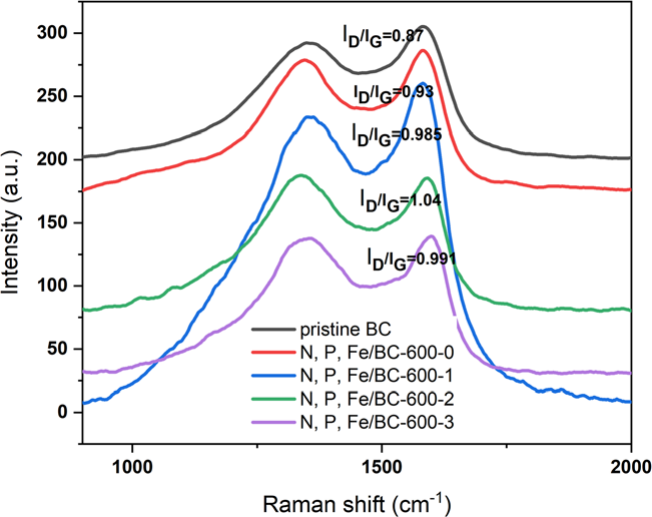
Raman spectra of pristine BC and N, P, Fe/BC-600-*n* (*n* = 0, 1, 2, 3).

As shown in Fig. 2, the Raman spectra for the biochar samples presented two key peaks at 1340 cm^−1^ and 1591 cm^−1^. These signals were attributed to the D band, signifying the presence of disordered carbon, and the G band, representing graphitic carbon, both typical features of carbon-based substances. The intensity of D and G bands (*I*_D_/*I*_G_) of pristine BC, N, P, Fe/BC-600-0, N, P, Fe/BC-600-1, N, P, Fe/BC-600-2, N, P, Fe/BC-600-3 were compared, and the results were listed in Fig. 2. The *I*_D_/*I*_G_ ratio for N, P, Fe/BC-600-0 was 0.93, which is greater than that of the original biochar (0.87). This indicates that phosphorus doping plays a role in enhancing the formation of edge defects within the porous carbon structures, a finding consistent with earlier studies.^37^ As the K_2_CO_3_/BC mass ratio increased, the *I*_D_/*I*_G_ ratio initially rose and subsequently declined, reaching its peak value (1.04) at an activator ratio of 2. N, P, Fe/BC-600-2, exhibiting a higher degree of structural disorder and defects, could offer a greater number of active sites, thereby potentially improving supercapacitor performance.^37,38^ The results above showed that the N, P, Fe/BC-600-2 sample possessed a superior specific surface area, a more significant micropore volume, plus a higher *I*_D_/*I*_G_ value. Such combined attributes strongly indicated the potential for better energy storage performance. These findings led to the identification of a K_2_CO_3_ to biochar mass ratio of 2 as the most favorable parameter examined in this work.

#### 3.1.2 The effect of pyrolysis temperature

**Fig. 3:**
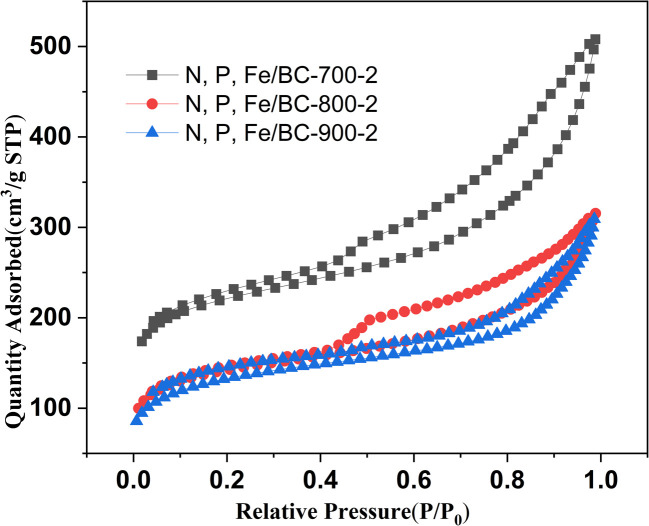
N_2_ adsorption–desorption isothermal curves of N, P, Fe/BC-*T*-2 (*T* = 700, 800, 900 K) at 77 K.

**Fig. 4:**
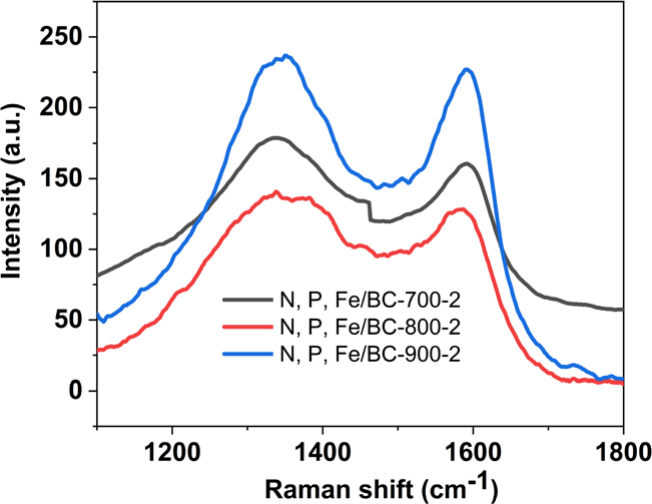
Raman spectra of samples pyrolyzed at different temperature.

The N_2_ adsorption–desorption isotherms of N, P, Fe/BC-700-2, N, P, Fe/BC-800-2 and N, P, Fe/BC-900-2 were shown in Fig. 3. Numerous mesopores were indicated in three of the analyzed samples, which showed a type IV isotherm along with H3-type hysteresis. The BET specific areas of N, P, Fe/BC-600-2 (Fig. 1), N, P, Fe/BC-700-2, N, P, Fe/BC-800-2 and N, P, Fe/BC-900-2 were calculated to 467.3, 824.5, 521.3, 482.1 m^2^ g^−1^, respectively. The total pore volume of N, P, Fe/BC-600-2 (Table 1), N, P, Fe/BC-700-2, N, P, Fe/BC-800-2 and N, P, Fe/BC-900-2 was 3.51, 7.88,4.892, 4.794 cm^3^, respectively. As the pyrolysis temperature was raised, leading to increased carbonization, the BET surface area of the synthesized materials initially expanded, peaking at 700 °C, before subsequently declining. Conversely, the N, P, Fe/BC-900-2 sample showed the smallest BET surface area and overall pore volume within the group. This reduction likely stemmed from heteroatom decomposition combined with pore structure collapse occurring at such high processing temperatures. The determined *I*_D_/*I*_G_ ratios were 1.04, 1.10, 1.05, 1.06, and 1.06 for N, P, Fe/BC-600-2, N, P, Fe/BC-700-2, N, P, Fe/BC-800-2, and N, P, Fe/BC-900-2, correspondingly. N, P, Fe/BC-700-2 exhibited the highest value, indicating the highest degree of defects (Fig. 4).

**Fig. 5:**
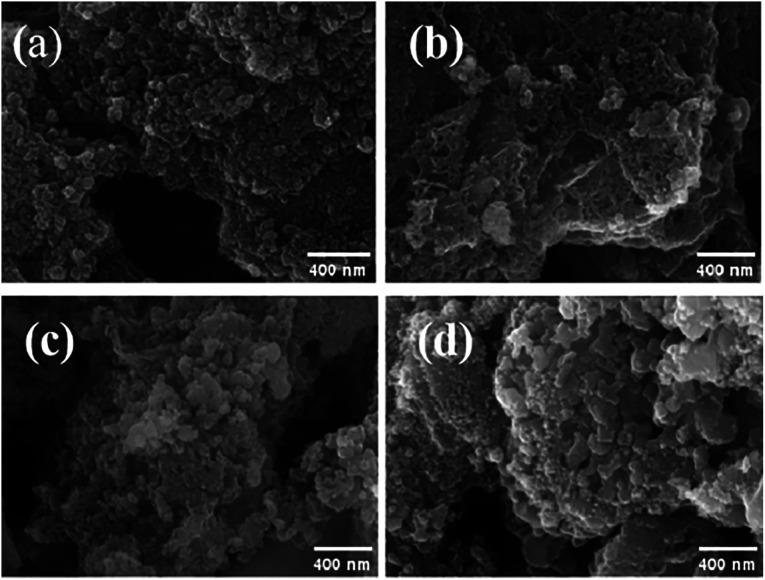
SEM images of N, P, Fe/BC-600-2 (a), N, P, Fe/BC-700-2 (b), N, P, Fe/BC-800-2 (c), and N, P, Fe/BC-900-2 (d).

The surface structures of N, P, Fe/BC-600-2, N, P, Fe/BC-700-2, N, P, Fe/BC-800-2, and N, P, Fe/BC-900-2 were observed in the SEM image (Fig. 5), which exhibited marked differences in their structural features. The N, P, Fe/BC-600-2 has rough surface with layered structure. The N, P, Fe/BC-700-2 showed a superb porous framework with a multitude of linked pores distributed across its surface. In contrast, N, P, Fe/BC-800-2 and N, P, Fe/BC-900-2 exhibited surface clusters. This is likely because higher temperatures can lead to the breakdown of the biochar's internal pore structure, a finding that aligns with the BET analysis.

**Fig. 6:**
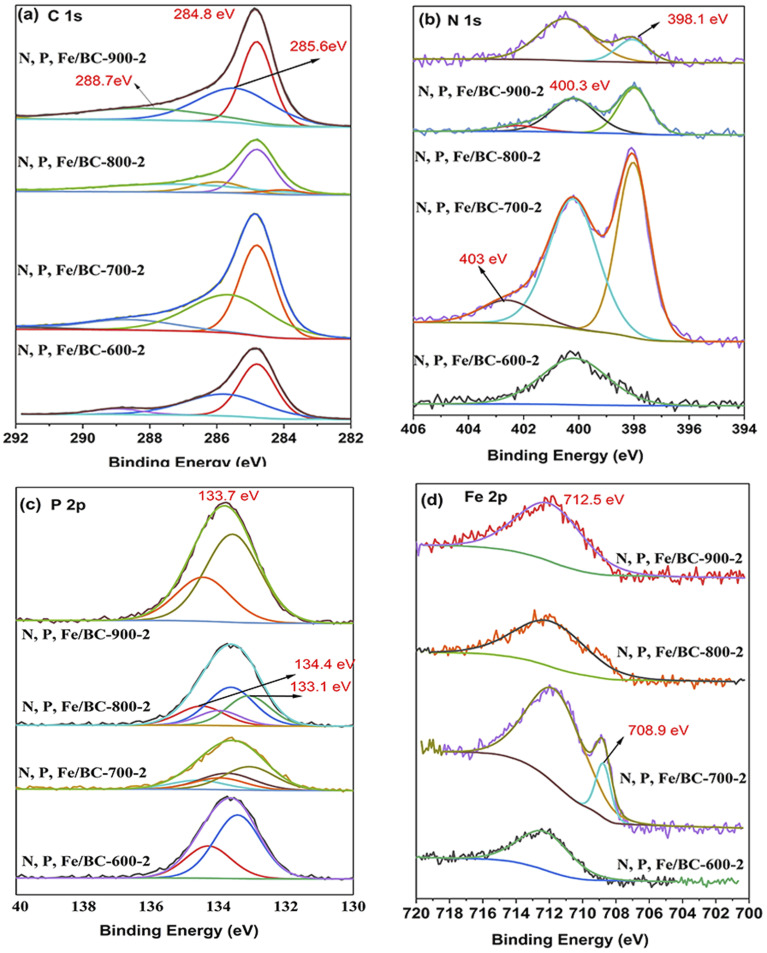
XPS spectra of N, P, Fe/BC-600-2, N, P, Fe/BC-700-2, N, P, Fe/BC-800-2, and N, P, Fe/BC-900-2. (a) C 1s, (b) N 1s, (C) P 2p, (d) Fe 2p.

The surface makeup and chemical bonding states of C, N, P, and Fe within the produced N, P, Fe/BC sample collection were analyzed using X-ray photoelectron spectroscopy (XPS). The full spectra of N, P, Fe/BC-600-2, N, P, Fe/BC-700-2, N, P, Fe/BC-800-2, and N, P, Fe/BC-900-2 were composed of C 1s, N 1s, P 2p and Fe 2p. The results of elemental analysis of C, N, P, Fe were illustrated in Table 2, indicating N, P, Fe were successfully incorporated into biochar. As the pyrolysis temperature was raised, we observed an increase in the proportions of both C and P. Conversely, the relative amounts of N and Fe tended to decrease under these higher temperature conditions. Fig. 6 displays the C 1s core-level spectra for the N, P, Fe/BC-600-2, N, P, Fe/BC-700-2, N, P, Fe/BC-800-2, and N, P, Fe/BC-900-2 materials.

**Table 2:** Elemental analysis of C, N, P, Fe of N, P, Fe/BC-700-2, N, P, Fe/BC-800-2, and N, P, Fe/BC-900-2

Samples	Elements	C	N	P	Fe
N, P, Fe/BC-600-2	Atomic%	85.45	9.96	2.65	1.94
N, P, Fe/BC-700-2	Atomic%	86.42	8.07	3.31	2.20
N, P, Fe/BC-800-2	Atomic%	87.84	4.11	3.64	4.41
N, P, Fe/BC-900-2	Atomic%	89.05	2.32	6.05	2.58

The C 1s spectrum revealed four main peaks at binding energies of 284.8, 285.7, 288.7 eV (Fig. 6a). These peaks are assigned to the following carbon functional groups: C=C, C–N, and C–O/C=N, respectively.^39^ This observation indicated the effective incorporation of nitrogen into the carbon framework. For N 1s, N, P, Fe/BC-600-2 had only one peak at 400.3 eV, which was related to pyrrolic N. In contrast, the remaining three samples displayed peaks at 398.1, 400.3, and 403.0 eV (Fig. 6b), corresponding to pyridinic N, pyrrolic N, and pyridine-*N*-oxide, respectively.^39,40^ One p-electron can be donated by pyridine-N to the π-conjugated system, which reportedly aids electrolyte movement and results in enhanced electrical conductivity. Whereas, the electron-donor nature of pyrrolic-N means it plays a vital part in facilitating electron transfer processes.^41^ Besides, pyridine-N oxide functions as an electron-accepting species during the charge–discharge cycles of the carbon electrode, which can lead to a decrease in ion transfer resistance.^40^Table 2 showed the relative surface concentration of nitrogen species. As the pyrolysis temperature was increased to 900 °C, the signals corresponding to pyridinic nitrogen and pyridine-N oxide (at 403 eV) vanished, likely due to their transformation into graphitic nitrogen.^40^ High-resolution P 2p spectra for N, P, Fe/BC-700-2, N, P, Fe/BC-800-2 exhibited peaks at 133.1, 133.4 and 133.7 eV (Fig. 6c), which were generated by P–C and C–O–P bonds.^42,43^ Meanwhile, the peaks of N, P, Fe/BC-600-2 and N, P, Fe/BC-900-2 were concentrated at the binding energies of 133.4 and 133.7 eV. The corresponding Fe 2p_3/2_ spectra were divided into two sections at 708 eV, and 712 eV (Fig. 6d), representing Fe^2+^, and Fe^3+^ states, respectively.^44^ This finding indicates a synergistic effect, suggesting that the combined doping strategy preferentially encourages the development of these specific configurations.

### 3.2 Supercapacitor performance

To gauge their suitability for supercapacitor applications, we examined the electrochemical characteristics of the synthesized materials. Cyclic voltammetry (CV), galvanostatic charge–discharge (GCD), and electrochemical impedance spectroscopy (EIS) were the analytical techniques employed for this evaluation. All experiments were performed using a three-electrode setup containing 6 M potassium hydroxide (KOH) as the aqueous electrolyte solution. Fig. 6a illustrates the cyclic voltammograms obtained at a scan rate of 30 mV s^−1^ within a potential window of 0 to 1.2 V for pristine biochar, N, P, Fe/BC-600-2, N, P, Fe/BC-700-2, N, P, Fe/BC-800-2, and N, P, Fe/BC-900-2. These curves exhibited a near-rectangular form, indicative of typical electrical double-layer capacitor behavior and a swift electrochemical reaction in these substances.^45^ Among all the samples, N, P, Fe/BC-700-2 exhibited the greatest discharging capacitance, which implied best supercapacitor performance. Fig. 6b presents the GCD profiles of the synthesized materials obtained at a current density of 1 A g^−1^. The GCD curves, remarkably, all showed a distinctly triangular form, indicating swift and efficiently reversible charge and discharge behavior.^44^ The specific capacitances of pristine biochar, N, P, Fe/BC-600-2, N, P, Fe/BC-700-2, N, P, Fe/BC-800-2, and N, P, Fe/BC-900-2 were 26.6, 73.3, 141.7, and 65.8 F g^−1^, respectively, using eqn (1). These results showed that the GCD and CV tests were consistent. N, P, Fe/BC-600-2 showed poor capacitive performance owing to both incomplete carbonization and insufficient heteroatom doping (only formed pyrrolic N and C–O–P bonds). In comparison to the other synthesized materials, N, P, Fe/BC-700-2 exhibited the most favorable supercapacitor performance. This suggested that the cooperative interaction among N, P, and Fe elements significantly enhanced the material's specific capacitance. To summarize, the notably improved supercapacitor performance of the N, P, Fe/BC-700-2 electrode can likely be ascribed to its maximal specific surface area, abundant mesoporous framework, presence of defect sites, and the optimized levels of incorporated heteroatoms. As illustrated in Fig. 3, the mesopore volume for the N, P, Fe/BC-700-2 sample was determined to be 7.88 cm^3^ g^−1^, significantly exceeding that of the other synthesized materials. It's understood that developing a mesoporous structure within the material can effectively speed up electrolyte ion movement and enhance electron transport pathways. Furthermore, the N, P, Fe/BC-700-2 sample stood out, possessing a relatively high pyridinic-N content (8.07 atomic%) compared to its counterparts. This specific nitrogen arrangement is considered a key contributor to its strong conductive performance. As previously indicated, the ultimate electrochemical capabilities result from an intricate interplay of various material properties.

**Fig. 7:**
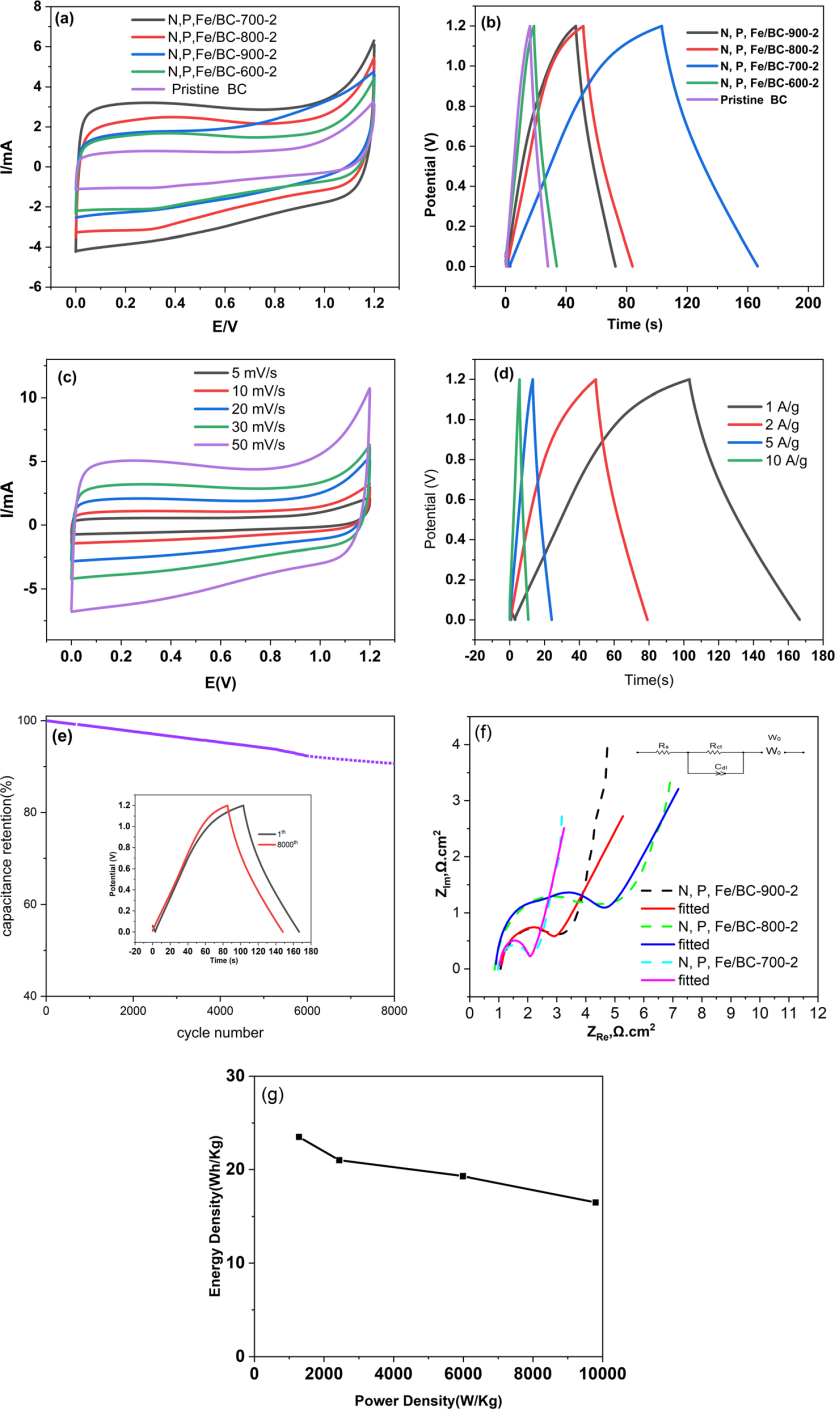
Electrochemical performance of N, P, Fe/BC samples: (a) CV curves at 30 mV s^−1^ in 6 M KOH. (b) GCD curves at 1 A g^−1^. (c) CV curves of N, P, Fe/BC-700-2 with scan rates from 10 to 50 mV s^−1^. (d) Specific capacitance of N, P, Fe/BC-700-2 at current densities ranging from 1 to 10 A g^−1^. (e) Cycling performance of N, P, Fe/BC-700-2 at 1 A g^−1^. (f) EIS curves (the equivalent circuit is shown in the inset). (g) Ragone plot.

For a more comprehensive assessment of the electrochemical characteristics of all synthesized materials, Fig. 6c illustrates the cyclic voltammetry (CV) responses of N, P, Fe/BC-700-2 at various sweep rates, while Fig. 6d showed galvanostatic charge–discharge (GCD) data for every material across varying applied currents. Fig. 6c displays the cyclic voltammetry curves for sample N, P, Fe/BC-700-2 obtained at varying scan rates (5 to 50 mV s^−1^). At lower sweep speeds, these plots approximate a rectangular form. However, increasing the scan rate introduces a distinct hump between 0.8 and 0.2 V onto the generally rectangular shape. This deviation is attributed to redox reactions involving the incorporated N, P, and Fe heteroatoms. Fig. 7d showed the specific capacitances of N, P, Fe/BC-700-2 measured across a range of current densities from 1 to 10 A g^−1^. The results revealed that all the obtained curves displayed symmetrical, nearly isosceles triangular forms, without substantial deviation. This observation reinforces the superior capacitive performance of the materials, even under conditions of high current loading. Higher applied currents make ion transport within the electrode structure the limiting factor, which consequently leads to lower observed capacitance values. Even with a higher current load of 10 A g^−1^, a specific capacitance of 99.1 F g^−1^ was still observed, indicating a 70.2% capacitance retention and thus satisfactory stability. The outcomes of 8000 charge–discharge cycles at 1 A g^−1^ are shown in Fig. 7e, displaying a 90.7% capacitance retention and therefore indicating encouraging electrochemical stability. To further probe the samples' electrochemical behavior, electrochemical impedance spectroscopy (EIS) was employed, examining their resistances and the electrode/electrolyte interface's frequency response. Fig. 7f presents the electrochemical impedance spectroscopy (EIS) plots, along with the fitted equivalent circuit (inset), for the synthesized samples. The measurements took place at the open-circuit voltage across a frequency range spanning from 10^−2^ to 10^5^ Hz, and ZSimpWin software was used to fit the equivalent circuit model. A near-vertical trace along the *Z*′ axis at lower frequencies, indicative of capacitive performance,^46^ contrasted with a semicircular feature observed in the higher frequency range, which corresponded to the charge transfer resistance (*R*_ct_). *R*_ct_ indicates the impedance to charge movement across the electrode–electrolyte interface. The intercept point on the real axis relates to the equivalent series resistance (*R*_s_).^47^ Fitting the impedance data to an equivalent circuit provided specific resistance values. Calculations showed *R*_s_/*R*_ct_ pairs as follows: 1.06/2.34 Ω for N, P-BC-700-2; 0.86/3.74 Ω concerning N, P-BC-800-2; and 0.97/1.23 Ω regarding N, P-BC-900-2. Achieving a lower solution resistance (*R*_s_) is beneficial because it minimizes energy dissipation during charge and discharge cycles. Likewise, a reduced charge transfer resistance (*R*_ct_) facilitates quicker diffusion and penetration by electrolyte ions, thereby enhancing the material's capacity to store energy effectively, especially when operating under high current demands.^48^ Above all, N, P-BC-700-2 showed the relative better electrochemical performance. Effective charge transport is facilitated by its large specific surface area and well-developed pore system, which explains this observation. Fig. 6g showed Ragone plot of N, P-BC-700-2, which exhibited the highest energy density of 23.5 Wh kg^−1^ at 1280 W kg^−1^ power density.

In order to evaluate the electrochemical performance of N, P, Fe/BC-700-2, the properties with those previously reported electrode materials based on biochar materials for supercapacitors were compared (Table 3). Although the specific capacitance of N, P, Fe/BC-700-2 was moderate, the utilization of surplus sauce-flavor liquor sludge to support “waste to wealth” strategy is the main advantage of our work.

**Table 3:** Electrochemical properties compared with those previously reported electrode materials based on biochar materials for supercapacitors

Feedstock	Sample	*S*_BET_ (m^2^ g^−1^)	Current density (A g^−1^)	Electrode system	Electrolyte	Specific capacitance (F g^−1^)	Ref.
Cornstalk	N, P co-doped biochar	793.99	1	Three-electrode	6 M KOH	203.5	4
				Two-electrode		77.78	
Mantis shrimp shell	N, S co-doped biochar	401	1	Three-electrode	6 M KOH	201	6
Potato starch	N, O co-doped biochar	2267	1	Three-electrode	1 M KOH	461	49
Lessonia trabeculata macroalgae	Activated biocarbon	769	1	Two-electrode	1 M KOH	81.6	50
Algae	N, O-codoped hierarchical porous carbons	2073	1	Two-electrode	EMIM BF_4_	130	51
Ascophyllum nodosum	Activated biocarbon	1493	0.5	Three-electrode	1 M KOH	207.3	52
Laminaria japonica	Porous carbon	2088.31	1	Two-electrode	6 M KOH	126	53
Flammulina velutipes	N, O co-doped biochar	1174.2	0.5	Three-electrode	1.0 M H_2_SO_4_	470.5	1
Excess sludge of sauce-flavor liquor	N, P, Fe co-doped biochar	824.5	1	Two-electrode	6 M KOH	141.7	This work

## 4 Conclusions

The valorization of waste materials, particularly industrial sludges, is a critical aspect of sustainable development. While diverse strategies exist for sludge management, ranging from advanced *in situ* characterization^54^ to chemical conditioning for improved dewaterability,^55^ our work demonstrates a direct and effective conversion pathway to fabricate a series of porous biochars co-doped with N, P, and Fe starting from surplus sauce-flavor liquor sludge. This study presents a straightforward and efficient pathway for creating a series of porous biochars co-doped with N, P, and Fe, starting from surplus sauce-flavor liquor sludge. The preparation involved tuning the potassium carbonate ratio and the pyrolysis temperature, which allowed control over introducing structural defects into the final materials. The findings suggested that substantial nitrogen and iron amounts could inherently incorporate into the biochar. Conversely, the phosphorus element was incorporated *via* the introduction of phytic acid, which additionally functioned as a pore-generating agent, contributing to the development of porous structures under high-temperature conditions. The optimized N, P, Fe/BC-700-2 material possesses a substantial specific surface area (824.5 m^2^ g^−1^), a wealth of mesopores, a significant nitrogen concentration (8.07 atomic%), suitable phosphorus (3.31 atomic%) and iron (2.2 atomic%) levels. Consequently, the synthesized N, P, Fe/BC-700-2 sample demonstrated a comparatively high capacitance of 141.7 F g^−1^ when tested at a current density of 1 A g^−1^ in a 6 M KOH electrolyte. After 8000 cycles, N, P, Fe/BC-700-2 maintained 90.7% of its original capacitance, demonstrating adequate cycling stability. This study illustrates that the strategic incorporation of N, P, and Fe elements can enhance supercapacitor performance. Beyond the electrochemical achievements, this research also highlights a key environmental co-benefit: the successful upcycling of brewery waste into functional materials. This approach reduces the environmental impact associated with sludge disposal, conserves resources by avoiding the use of virgin materials, and aligns with the growing global demand for sustainable manufacturing and circular economy solutions.

## Author contributions

Suxing Luo: conceptualization, methodology, validation, writing – original draft preparation, project administration, funding acquisition. Jiang Li: writing – review & editing, data curation, conceptualization, supervision, funding acquisition. Meizhi Yang: writing – review & editing, validation, methodology. Ping Zeng: writing – original draft preparation, supervision, funding acquisition. Yuanhui Wu: data curation, supervision, funding acquisition.

## Conflicts of interest

No conflicts of interest.

## Data Availability

The data generated or used in this study are included in the submitted article. Additional data used for the study are available from the corresponding authors upon reasonable request.
